# Investigating the cognitive architecture of verbal fluency: evidence from an interference design on 487 controls

**DOI:** 10.3389/fpsyg.2024.1441023

**Published:** 2024-12-16

**Authors:** Flore Dorchies, Camille Muchembled, Corinne Adamkiewicz, Olivier Godefroy, Martine Roussel

**Affiliations:** ^1^Laboratory of Functional Neurosciences (UR UPJV 4559), Jules Verne University of Picardie, Amiens, France; ^2^Department of Speech Therapy, Jules Verne University of Picardie, Amiens, France; ^3^Department of Neurology, Amiens University Hospital, Amiens, France

**Keywords:** verbal fluency, dual tasks, linguistic processes, executive processes, switching, time course, cognitive architecture, healthy participants

## Abstract

**Introduction:**

Numerous studies have explored the linguistic and executive processes underlying verbal fluency using association designs, which provide limited evidence. To assess the validity of our model, we aimed to refine the cognitive architecture of verbal fluency using an interference design.

**Methods:**

A total of 487 healthy participants performed letter and semantic fluency tests under the single condition and dual conditions while concurrently performing a secondary task that interferes with speed, semantics, phonology, or flexibility. We examined the effect of such interference on fluency indices including correct responses, clustering, switching, and time course.

**Results:**

(1) All secondary tasks decreased fluency (*p* < 0.0001, all), (2) including a simple concurrent task that solely engages the attentional activation system (i.e., speed interference) and (3) a complex concurrent task that affects the ability to alternate (i.e., flexibility interference). (4) Linguistic secondary tasks (which engage phonological and semantic processes, in addition to attention) led to a greater decrease in fluency than speed interference (*p* < 0.0001), (5) with a more pronounced decrease in semantic fluency induced by semantic interference (*p* < 0.0001), and (6) the highest decrease in all types of fluency induced by phonological interference (*p* < 0.0001). In terms of derived indices, (7) speed interference decreased switching without affecting clustering (*p* < 0.0001) and (8) phonological interference mainly affected the first time interval, whereas speed and flexibility interference primarily affected the last time interval (*p* < 0.0001, all).

**Discussion:**

These results, based on an interference design, indicate that letter and semantic fluency involve output lexico-phonological and semantic processes with which the strategic search process interacts, as well as an attentional component necessary to accelerate overall processing. These results also highlight interactions with other executive processes, such as those involved in stimulus dimension alternation, which require further analysis. They support our model and provide information concerning derived indices. The commonly claimed associations of executive function with switching and of semantic ability with clustering are only partially supported by our results. Finally, word production appears to be modulated by different cognitive processes over time, with a prominence of the phonological output lexicon in early production and more demanding processing (i.e., executive functioning) in late production.

## 1 Introduction

Verbal fluency tests are among the most commonly used neuropsychological assessment tools due to their simple and rapid administration and sensitivity to aging and brain diseases (Godefroy et al., 2018; Henry et al., 2004; Henry and Crawford, 2004; Thiele et al., 2016). Although they have been extensively studied, the respective contributions of different cognitive processes, such as the linguistic and executive components, are still unclear. In this context, evaluating the number of correct words as the only measure does not fully capture the processes that underly verbal fluency (Thiele et al., 2016; Troyer et al., 1997).

To identify the contribution of linguistic and executive processes in fluency tasks, two approaches, sometimes associated, have been developed: (1) the study of additional indices that can be extracted from verbal fluency tasks (i.e., derived indices) and (2) the study of the relationships between fluency tasks and other tests assessing language and executive functions.

The first approach is based on derived indices, such as errors, clusters, and the time course of production. The main types of errors include perseverations (i.e., inappropriately repeated words) and rule-break errors (i.e., words not corresponding to the cue) (Itaguchi et al., 2022; Liampas et al., 2022; Wajman and Cecchini, 2023). They can be observed in certain neurological diseases (Cipolotti et al., 2020; Itaguchi et al., 2022; Shao et al., 2014). The perseveration rate has been shown to be associated with impaired working memory (Azuma, 2004; Rosen and Engle, 1997). Rule-break errors have been attributed to impaired inhibition (Cipolotti et al., 2020). Other derived indices include clustering (production of consecutive words within the same letter or semantic subcategories) and switching scores (corresponding to shifting between subcategories) (Troyer et al., 1997). According to the prevailing view, the mean cluster size is attributed to lexico-semantic processes (Bose et al., 2022; Faroqi-Shah and Milman, 2018; Raboutet et al., 2010; Tröster et al., 1998; Troyer et al., 1997, 1998) and the ability to switch between subcategories is attributed to executive processes (Bose et al., 2017, 2022; Troyer et al., 1997, 1998). This interpretation has both found support and has been challenged in neuropsychological studies (Abwender et al., 2001; Bose et al., 2022; Dorchies et al., 2024; Mayr, 2002). Most studies have been based on association designs (i.e., designs based on the association between indices and external measures of linguistic and executive processes), which provide only a limited level of evidence relative to interventional designs.^1^ Few studies have used an interventional design based on the dual-task paradigm, which provides a higher level of evidence. Using a secondary tapping task (which presumably involves the frontal lobe). Troyer et al. (1997) showed a dual-task decrease in switching but not clustering. Given these limitations, the precise processes measured by these indices require supplementary evidence. Time course analysis of word production has consistently demonstrated a decrease over time (Bose et al., 2017, 2022; Crowe, 1998; Demetriou and Holtzer, 2017; Itaguchi et al., 2022; Kassir et al., 2023; Luo et al., 2010; Michalko et al., 2023; Raboutet et al., 2010), but the cognitive interpretation of this trend remains debated (Thiele et al., 2016). In regard, to the preponderant role of linguistic processes, the model proposed by Mayr and Kliegl (2000) posits a constant involvement of executive processes responsible for initiating and sustaining word retrieval, while semantic processes become increasingly engaged as the task progresses. This was supported by Raboutet et al. (2010), who observed an increase in semantic indices over time. A third study focusing on bilinguals (Kassir et al., 2023) interpreted the greater decline over time in the nondominant language as evidence for the prominent role of lexico-semantic access (i.e., vocabulary) as time goes on. Conversely, other studies suggest that linguistic processes dominate early in the task, with executive processes becoming more involved as it progresses (Bose et al., 2022; Friesen et al., 2015; Michalko et al., 2023; Shao et al., 2014). Regression analyses have shown that vocabulary is critical for initial production, while updating abilities and lexical access speed are more involved in final production (Shao et al., 2014). Bose et al. (2022) found a greater temporal decline in monolinguals compared to bilinguals, who were assumed to have better executive control. Michalko et al. (2023) link the decline in word production to reduced typicality and semantic similarity, as well as low executive capacity, suggesting that word retrieval becomes less spontaneous and more cognitively demanding over time. To summarize, the performance patterns over time have been interpreted in three studies as evidence for the predominant involvement of linguistic processes (Kassir et al., 2023; Mayr and Kliegl, 2000; Raboutet et al., 2010), and in four studies as evidence for the predominant involvement of executive processes (Bose et al., 2022; Friesen et al., 2015; Michalko et al., 2023; Shao et al., 2014). Given these inconsistencies and the limited evidence provided by association designs, additional studies are needed to clarify the underlying processes that drive the time course of fluency production.

A second approach has established a set of relationships between verbal fluency, language, and executive abilities. It has documented an association between verbal fluency and linguistic abilities (naming, lexical access, vocabulary size) (Carpenter et al., 2020; Dorchies et al., 2024; Godefroy et al., 2023; Gonzalez-Burgos et al., 2019; Kassir et al., 2023; Kraan et al., 2013; Shao et al., 2014; Whiteside et al., 2016), processing speed (Godefroy et al., 2023; Gonzalez-Burgos et al., 2019; Kraan et al., 2013; McDowd et al., 2011), and executive functioning (e.g., inhibition, working memory, updating) (Bose et al., 2022; Gonzalez-Burgos et al., 2019; Kraan et al., 2013; McDowd et al., 2011; Patra et al., 2020; Shao et al., 2014). The strength of the association has been shown to vary depending on the type of fluency, with a frequently higher association between letter fluency and executive abilities and between semantic fluency and linguistic abilities (Bose et al., 2022; Faroqi-Shah and Milman, 2018; Friesen et al., 2015; Kraan et al., 2013; Luo et al., 2010; Patra et al., 2020). Several studies used a dual-task design to manipulate specific components of fluency. Martin et al. (1994) reported a double dissociation, with a greater decrease in letter fluency using a secondary motor task (digital tapping), presumably involving executive control supported by frontal regions, and a greater decrease in semantic fluency using a secondary object decision task, presumably involving semantic processing supported by temporal regions. Moscovitch (1994) and Troyer et al. (1997) replicated this greater decrease in letter fluency with the digital tapping task, confirming that this secondary task interferes with frontal-lobe functioning. Rende et al. (2002) employed three secondary tasks (i.e., articulatory suppression, cube comparison, and arithmetic switching) to manipulate the three main components of working memory: the phonological loop, visuospatial sketchpad and an aspect of central executive functioning (i.e., set shifting). Their results suggest that phonological loop and visuospatial sketchpad significantly contribute to fluency performance - primarily letter fluency for the phonological loop and semantic fluency for the visuospatial sketchpad - while the executive component is equally involved in both types of fluency. More recently, Fournet et al. (2021) investigated two secondary tasks, one involving processing speed (simple reaction time) and the other inhibition (Go/No-go). They observed a dual-task cost for both fluency and non-verbal tasks, suggesting that these secondary tasks compete for the same attentional resources required for fluency. Although none of these secondary tasks can be considered completely process-pure (i.e., interfering only with the specific fluency component without engaging other processes), the extent to which they equally or differentially disrupt fluency performance provides valuable insights into the processes underlying letter and semantic fluency.

Finally, the set of relationships between processes led to studies that proposed and validated a model of verbal fluency (Godefroy et al., 2023, 2024; Kassir et al., 2023). It is based on the model of Ellis and Young (1988), which specified the linguistic processes involved in oral language production: semantic memory, an output lexicon, a phonological-assembly buffer, and the articulation of language. The model of Godefroy et al. (2023) hypothesized that the fluency task additionally triggers two control processes required for a rapid strategic search: (1) a specific strategic search process, which selects and activates words from an output lexicon and possibly from a semantic memory lexicon and (2) a general attentional activation process required to accelerate the processing speed (Godefroy et al., 2010). According to this model, performing a verbal fluency task involves three main types of processes operating in common: (i) linguistic processes, namely semantic and lexicophonological output processes (common to externally triggered oral expressions, such as those involved in the naming task), (ii) a general attentional activation process that optimizes processing speed (purposely assessed in a simple non-verbal task like the Trail Making Test part A, TMT A), and (iii) a strategic (i.e., cue-based, unusual) search process, which is not directly assessable (Godefroy et al., 2023). Prior to this proposal, no study has defined a functional architecture of executive processes and their relationships with other functions that might account for verbal fluency task. In particular, the interaction between strategic lexico-semantic search and semantic and lexico-phonological processes remained undefined. This model suggests that verbal fluency relies on two control processes (strategic search and attention) operating on semantic and lexico-phonological output processes. This functional architecture has been cross-validated in a study of bilingual Lebanese individuals, which supports the involvement of a single, centralized strategic search process, regardless of language or level of fluency (Kassir et al., 2023), and in a study of more than 2000 multicultural stroke patients from the MetaVCI map consortium (Weaver et al., 2021), which confirms the involvement of the three above-mentioned sets of processes (Godefroy et al., 2024).

Overall, the identification of linguistic and executive processes underlying the overall number of correct responses and errors, clustering and switching indices, and time-course of production is still based on limited evidence for both types of fluency (letter and semantic). Most studies have been based on an association design, which provides a limited level of evidence. The few existing studies using a dual-task design were constrained by a limited sample size (approximately 20 participants), and most secondary tasks were designed to interfere with executive processes, with little assessment of linguistic processes.

In order to assess the validity of our model (Godefroy et al., 2023), our main objective was to determine, the relative contribution of linguistic and executive processes in letter and semantic fluency using dual tasks. A new design consisting of four secondary tasks to engage executive (i.e., speed and flexibility) and linguistic (i.e., phonological and semantic) processes was expected to contribute to the elucidation of the cognitive processes that underly verbal fluency. The secondary goals were to identify the prominent processes indexed by the derived indices, cluster size, number of switches, and the time course of word production.

## 2 Population and methods

### 2.1 Population

We included 487 French-speaking participants aged over 40 years who could read, write, and count to 36 and knew the alphabet. The exclusion criteria corresponded to all conditions related to a pathology that could interfere with cognition (detailed in Supplementary Material). Based on our previous studies (Godefroy et al., 2014a; Godefroy et al., 2014b), the sample size was calculated to achieve a 95% confidence interval with a 2% margin around the fifth percentile. This calculation determined a target of 455 participants, which was exceeded with a final sample size of 487 participants.

In addition, we computed the analyses of Troyer et al. (1997) (i.e., clustering and switching indices, see 2.2.1) for the first 310 included participants.

The demographic and neuropsychological characteristics of all participants (*n* = 487) and of the Troyer subgroup (*n* = 310) are presented in Table 1. This subgroup did not differ from the total group in terms of demographic data. The order in which the tasks were performed was counterbalanced and did not differ between the two groups.

**Table 1:** Demographic and neuropsychological characteristics of participants.

		All (*n* = 487)	Troyer (*n* = 310)	Comparisons (*p*)
Age	(m ± sd)	61.6 ± 11.5	61.6 ± 11.4	0.96
Female	(%)	61	62.3	0.49
Handedness R/L/TL/A	(%)	89.9/5.3/1.8/2.9	89.4/5.2/2.3/3.2	
Schooling (years)	(m ± sd)	12.6 ± 3.3	12.6 ± 3.3	0.92
MMSEadj	(m ± sd) /30	28.5 ± 1.3	28.6 ± 1.2	0.04

Expressed as means ± standard deviations or percentages (%). R, right-handed; L, left-handed; TL, thwarted left-handed; A, ambidextrous; MMSEadj, Mini-Mental State Examination adjusted for education. Group comparisons were conducted using t-tests, Levene’s test and Fisher’s exact test.

The study was approved by the regional investigational review board (Comité d’Ethique pour les Recherches Non-Interventionnelles, Université de Picardie Jules Verne, Amiens, France; reference: 2022-06-1/2022–15).

### 2.2 Cognitive assessment

**Figure 1:**
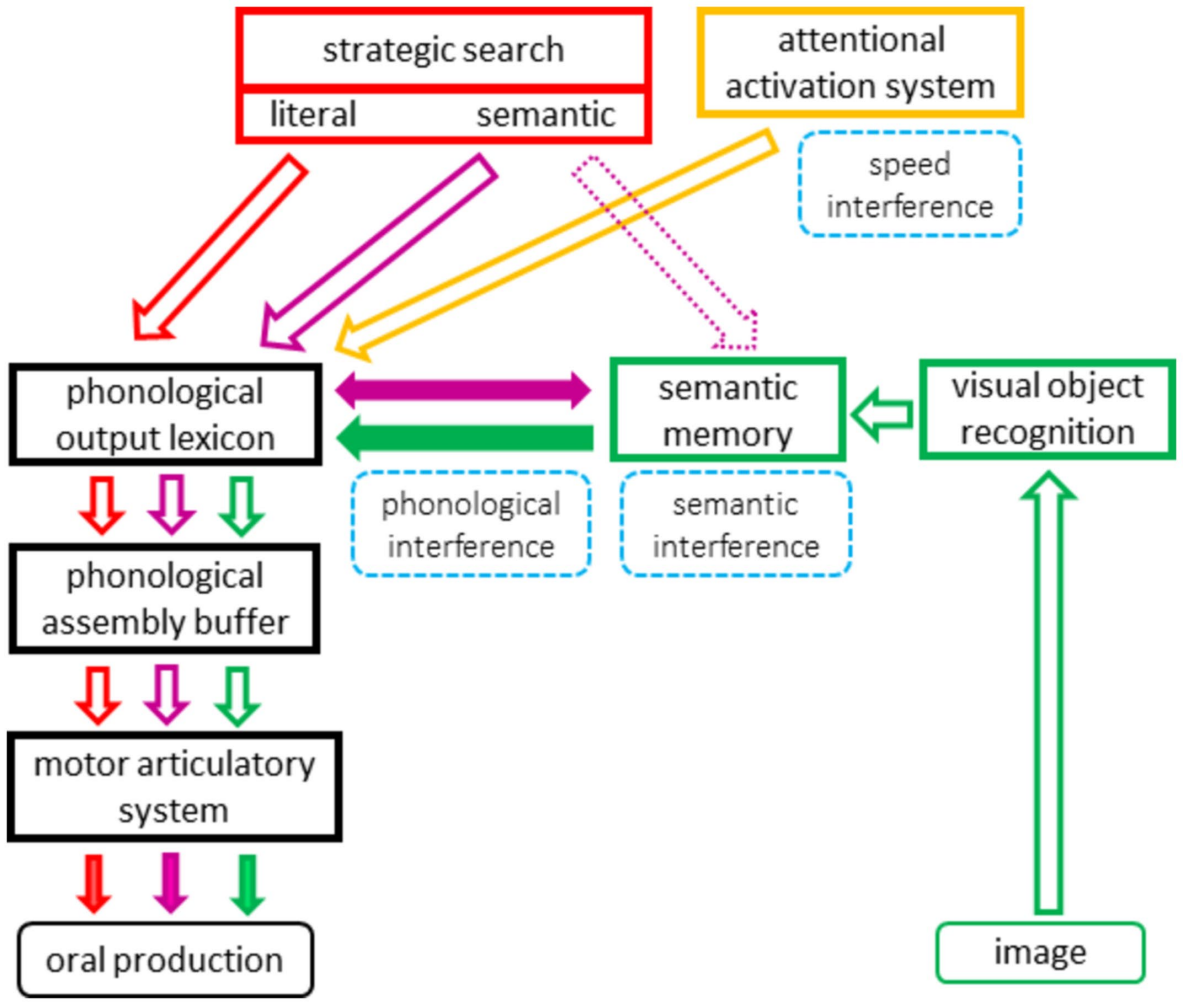
An adaptation of the model proposed by Godefroy et al. (2023). According to this model, fluency performance is related to the interaction of three sets of processes: (1) lexico-semantic storage and oral language output processes, which are also involved in confrontation naming (green arrows), (2) a strategic search process selecting and activating words from an output lexicon for letter fluency (red arrows) and possibly from a semantic memory-lexicon for semantic fluency (pink arrows), and (3) an attentional activation system that optimizes processing speed (yellow arrows). Secondary tasks (surrounded by dotted lines) were added and are located next to the processes they manipulate (framed by full lines).

Cognitive performance was assessed using verbal fluency tests under (i) single and (ii) dual conditions. Verbal fluency tests included letter (“p”) and semantic (animals) fluency. The dual-task paradigm included four secondary tasks that interfere with key processes involved in verbal fluency: executive interference that separately manipulated attention and flexibility and linguistic interference that separately manipulated semantic and phonological processes (Figure 1). For dual-task conditions, participants were instructed that both tasks had to be performed simultaneously and were of equal importance. Both fluency and interference were counterbalanced across subjects (detailed in Supplementary Figure A). The evaluation lasted 1 h.

#### 2.2.1 Verbal fluency tests

Letter and semantic fluency tests were performed in 1 min (Cardebat et al., 1990; Roussel and Godefroy, 2016). In the letter fluency test, participants were instructed to produce words beginning with the letter “p.” In French, the distinction between letter and phonological fluency is necessary because some letters correspond to multiple phonemes [e.g., the letter “p” can be associated with both /p/ and /f/, as in “parapluie” (“umbrella”) and “pharmacie” (“pharmacy”)]. In the semantic fluency test, all words produced had to belong to the category of animals.

The examiner wrote each produced word to permit further analysis. Following the GREFEX guidelines (Godefroy and Grefex, 2008), participants were instructed not to produce proper names, repetitions (even with different suffixes), or nonwords.

The total number of correct words produced in 1 min was counted, while excluding breaking rules (e.g., proper nouns like “Paris,” words not starting with “p” such as “tomato,” or words outside the animal category, such as “dress”), repetitions [e.g., “dog, (…), dog”], derivatives (e.g., if the participant says “partner, partnership,” only “partner” is counted), and superordinates (e.g., in “bird, blackbird, pigeon, sparrow,” “bird” is excluded as a superordinate term).

The progression of word production over time was analyzed by computing the percentage of correct words produced in four intervals: 0–15″, 16–30″, 31–45″, and 46–60″.

For the first 310 participants, clustering and switching indices were also calculated using the method proposed by Troyer et al. (1997). Clustering was defined as the mean cluster size, calculated by dividing the total cluster size by the number of clusters. Switching corresponded to the number of transitions between clusters. For letter fluency, clusters were made up of phonologically related words [e.g., words beginning with the same first two letters: “panier, parapluie, partir” (basket, umbrella, leave), or the same first two sounds: “pirate, pyramide” (pirate, pyramid), words differing by a vowel sound: “poule, pull” (hen, sweater), rhyming words: “pommier, poirier” (apple tree, pear tree), or a homonyms if explicitly distinguished by the participant or spelled out: “porc, port” (pig, port)]. When adapting clustering criteria to French, we disregarded accent variations. For semantic fluency, clusters were made up of animals within the same subcategory: forest animals (e.g., squirrel, hedgehog, badger…), farm animals (e.g., cow, horse, pig…), pets (e.g., cat, dog, hamster…), exotic animals (e.g., lion, antelope, rhinoceros…), aquatic animals (e.g., whale, dolphin, shark…), insects and arachnids (e.g., spider, woodlouse, ant…) and birds (e.g., swan, titmouse, blackbird…). If clusters overlapped, only the largest cluster was considered. Repetitions and errors were included in determining clusters and switches, as they provide insights into the lexical retrieval process during verbal fluency tasks (Troyer et al., 1997).

Thus, four scores were obtained for each fluency test: (1) the total number of correct words (the sum of all words produced, excluding errors), (2) production across time intervals 0–15″, 16–30″, 31–45″, and 46–60″ (the percentage of correct words produced in each interval), (3) mean cluster size (the sum of the cluster size divided by the number of clusters), and (4) number of switches (the number of transitions between clusters) (Troyer et al., 1997). The first and the second scores were computed for all participants (*n* = 487), while the third and fourth scores were computed for the first 310 participants.

#### 2.2.2 Visuomotor interfering tasks

**Figure 2:**
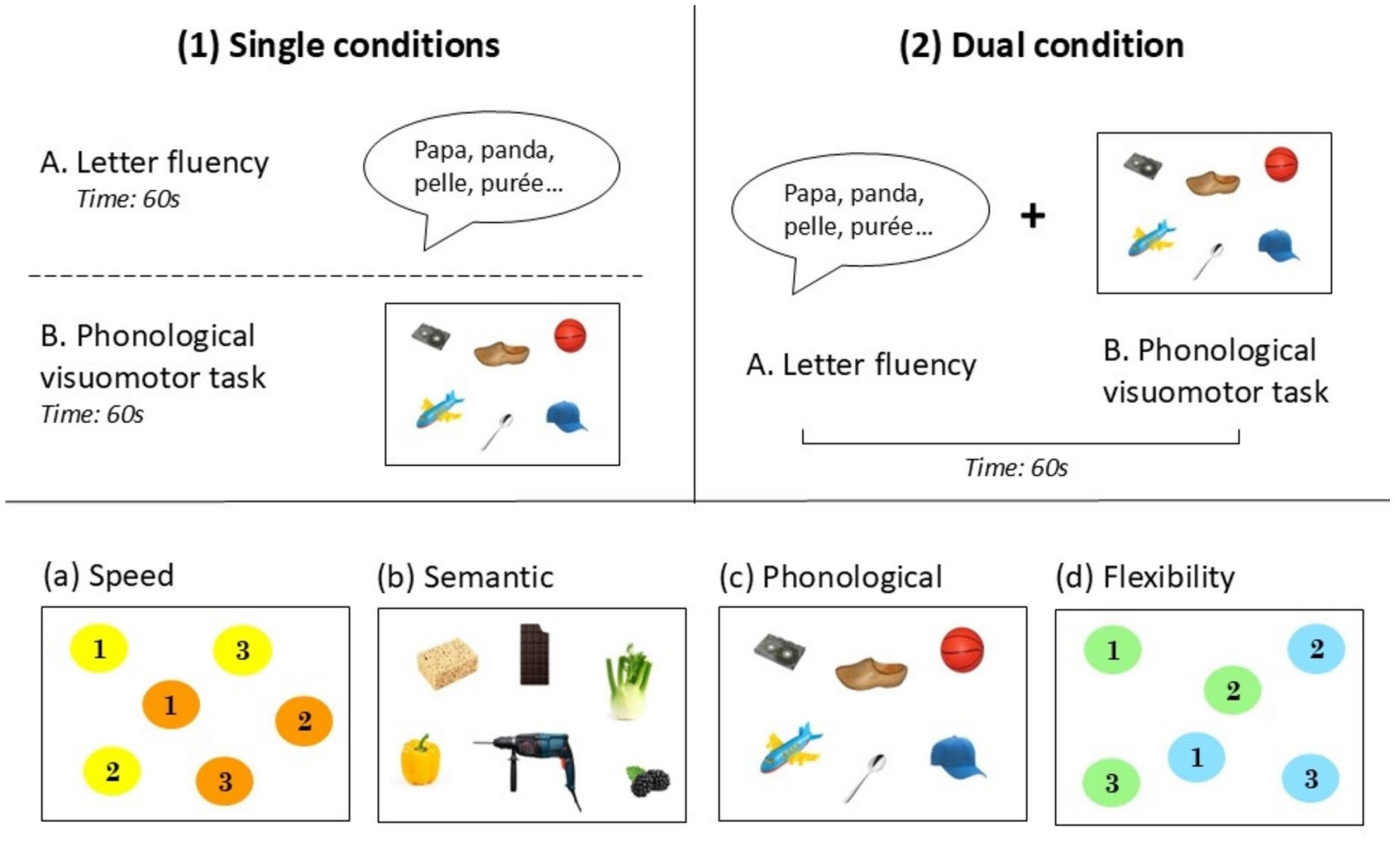
*Top of the figure:* Illustration of the dual-task paradigm procedure with examples of the letter “P” fluency and the phonological interference: (1) single conditions: **(A)** letter fluency alone [leading to the production of the words “papa, panda, pelle, purée…” (dad, panda, shovel, mashed potatoes…)] and **(B)** phonological visuomotor interfering task alone and (2) dual condition: letter fluency and phonological visuomotor interfering task simultaneously (A + B). *Bottom of the figure:* Illustrations of items from the four visuomotor interfering tasks: **(a)** speed (circle yellow numbers in ascending order), **(b)** semantic (circle vegetables, e.g., pepper and tools, e.g., drill), **(c)** phonological [circle words ending with [Ꜫt] e.g., “casquette” (cap) or [ɔ̃], e.g., “avion” (plane)], and **(d)** flexibility (circle numbers in ascending order and alternating colors, e.g., 1 green, 2 blue, 3 green…).

We examined the effects of secondary tasks that prominently manipulate linguistic or executive processes on verbal fluency performance. Interfering tasks used for the dual condition were visuomotor tasks adapted from the classical trail-making test (TMT) (Reitan, 1958; Roussel and Godefroy, 2008) and the color TMT test (D’Elia et al., 1996). We chose four types of interference: (1) speed, (2) semantic, (3) phonological, and (4) flexibility. Speed, semantic, and phonological interference were designed to engage processes involved in verbal fluency (i.e., located within the model), whereas flexibility interference was designed to involve an additional executive process that is presumably located outside of the verbal fluency model (Figure 1). Each task (fluency and visuomotor interfering tasks) was first performed individually (single condition) and then simultaneously (dual condition) (Figure 2 and Supplementary Figure A). The presentation format was similar to that of the TMT (A4 size). For visuomotor interfering tasks, we measured the number of correct circled responses (CR).

##### 2.2.2.1 Interference involving prominently executive processes

These tests were adapted from the color TMT (D’Elia et al., 1996). They used a procedure similar to that of the color TMT parts A and B, except for the time limitation (1 min), the number of items (72 instead of 25), and the trace (a circle instead of a link).

The speed interference task consisted of circling numbers of the same color in ascending order as quickly as possible (Figure 2A). This task was designed to prominently engage the attentional activation system, which is one of the processes involved in performing the verbal fluency task according to the model (Figure 1).

The flexibility interference task also consisted of circling numbers in ascending order, but this time alternating colors (1 green, 2 blue, 3 green…), as quickly as possible (Figure 2D). This task was designed to prominently engage the flexibility process, which is not involved in verbal fluency (Godefroy et al., 2023, 2024) (Figure 1).

##### 2.2.2.2 Interference involving prominently linguistic processes

The semantic interference task consisted of circling colored pictures that represent objects belonging to the same semantic category (vegetables and tools) as quickly as possible (Figure 2B). These 36 target items were mixed with 36 distractors (i.e., pictures with words that did not belong to the same semantic category). We controlled visual appearance (e.g., varying the color of vegetables) to avoid the use of visual cues that would allow the performance of the task without accessing semantic representation. In addition, each vegetable was matched to a food and each tool to an object. Each pair of target items and each distractor had a similar lexical frequency. This task was designed to prominently engage semantic memory (through visual and non-verbal access)—a process located within the verbal fluency model (Figure 1).

The phonological interference consisted of circling colored pictures that represent words ending with the same phonemes [Ꜫt] and [ɔ̃] in French [e.g., “casquette” (cap) and “avion” (plane)] as quickly as possible (Figure 2C). These 36 target items were mixed with 36 distractors (i.e., pictures with words that did not have the same phonological ending). Each target item was matched in terms of lexical frequency and syllabic length with a distractor. This task was designed to prominently engage the phonological output lexicon, located within the verbal fluency model (Figure 1).

### 2.3 Main hypotheses

In terms of the effect of interference on the total number of correct responses, we expected that: (1) a simple concurrent task that only engages the attentional activation system (speed interference) would mildly decrease both letter and semantic fluency equally; (2) concurrent linguistic tasks that use attention and additional linguistic processes, such as phonological or semantic (semantic and phonological interference) processes, would affect verbal fluency more than the speed interference; (3) a concurrent task that mainly engages semantic memory through visual access (semantic interference) would have a greater impact on semantic fluency than letter fluency; (4) a concurrent task that primarily engages the phonological output lexicon and more generally, the sequential chain of oral production (phonological interference), should have a major impact on both fluency tasks; and (5) we had no prediction concerning the concurrent task that interferes, in addition to attention, with the flexibility executive process, which is not involved in fluency tasks (flexibility interference).

In addition, according to the common view that switching reflects executive processes and clustering linguistic processes, we also hypothesized that interference that prominently engages linguistic processes (i.e., phonological and semantic interference) would mainly affect clustering, whereas interference that prominently engages executive processes (i.e., speed and flexibility interference) would mainly affect switching. Finally, we had no hypothesis concerning uncertainties about the effect of executive and linguistic processes on the time course of fluency production.

### 2.4 Statistical analyses

#### 2.4.1 Prerequisite analyses

We first checked that the order of administration of visuomotor interfering tasks did not affect performance. As this was the case, it is not detailed. Second, after ensuring that the effect of the dual task on correct responses was significant for all types of interference (see below), we calculated the percentage decrease (dual condition minus single condition) for all performance scores, herein referred to as the decrease in correct responses. This resulted in four types of decreased correct responses for each interference, thus representing a total of 16 measurements [2 tasks (fluency and visuomotor interfering task) × 2 types of fluency (letter, semantic) × 4 types of interference (speed, semantic, phonological, flexibility)].

#### 2.4.2 Effect of interference on correct responses

The effect of interference on both fluency and visuomotor interfering tasks was first analyzed by repeated measures of ANOVA, with the within-subject factors of task (fluency, visuomotor interfering), fluency (letter, semantic), and type of interference (speed, semantic, phonological, flexibility). As this analysis primarily aimed to control for potential trade-offs between tasks across interference conditions, it focused on the main effects and the interaction of interest, namely the task × interference interaction. *Post-hoc* analyses were performed using simple contrasts. The effect of the four types of interference on fluency reduction was further examined by repeated ANOVA, with the within-subject factors of fluency (letter, semantic) and type of interference (speed, semantic, phonological, flexibility).

#### 2.4.3 Effect of interference on clustering and switching

The effect of the four types of interference on clustering and switching was analyzed by two repeated ANOVAs (first: cluster size, second: number of switches) with the within-subject factors of fluency (letter, semantic) and type of interference (single condition, speed, semantic, phonological, flexibility).

The effects of interference were compared using two series of simple contrast analyses with the single condition as the reference for the dual conditions (to examine the dual-task effect) and speed interference as the reference for the other types of interference (to examine the additional effect of semantic, phonological, and flexibility loading). As these were repeated contrast analyses, only *p* ≤ 0.01 were considered significant.

#### 2.4.4 Effect of interference on the time course

The effect of the four types of interference on the time course of production deliberately focused on the first and last time intervals (0–15″, 46–60″), which proved to be the most informative (Kassir et al., 2023). It was analyzed by repeated ANOVA with the within-subject factors of the time interval (0–15″, 46–60″), fluency (letter, semantic), and type of interference (single condition, speed, semantic, phonological, flexibility). The effects of interference were compared using simple contrast analyses.

Statistical analyses were conducted using Statistical Package for the Social Sciences (SPSS, version 26) software. Values with *p* ≤ 0.05 were considered significant unless otherwise indicated.

## 3 Results

### 3.1 Effect of interference on correct responses

**Figure 3:**
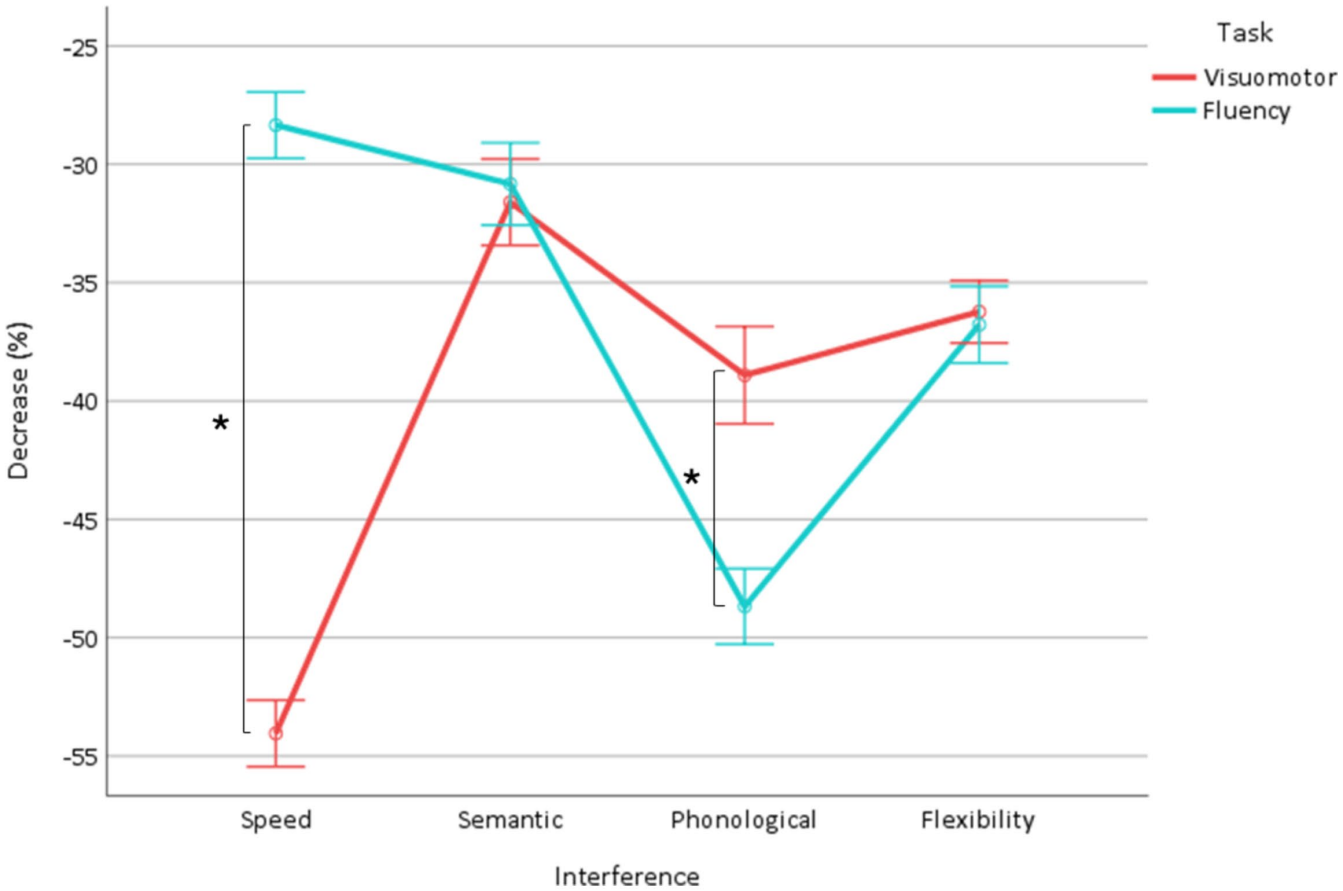
Decrease in fluency and visuomotor interfering tasks as a function of interference (speed, semantic, phonological, flexibility). *Indicate significant interactions.

The ANOVA showed (1) a task effect (*p* < 0.0001) due to a greater decrease in visuomotor interfering tasks (visuomotor interfering: −40.1 ± 0.5, fluency: −36.4 ± 0.7), (2) a fluency effect (*p* = 0.009) due to a slightly greater decrease in semantic fluency (letter: −37.5 ± 0.5, semantic: −38.9 ± 0.4), (3) an interference effect (*p* < 0.0001) due to a greater decrease in the phonological (−43.9 ± 0.6), then speed (−41.3 ± 0.5), then flexibility (−36.6 ± 0.5), and then semantic interference (−31.2 ± 0.5) (*p* < 0.0001, all), and (4) a task × interference interaction (*p* < 0.0001, Figure 3 and Table 2) due to a greater decrease induced by speed interference in the visuomotor interfering task and a greater decrease induced by phonological interference in the fluency task (*p* < 0.0001, both), whereas semantic and flexibility interference induced a similar decrease in both tasks (*p* = 0.3). This is illustrated in a graph plotting the decrease in fluency against that of the visuomotor interfering task (Godefroy et al., 1999) (Supplementary Figure B).

**Table 2:** Mean (± standard deviation) of verbal fluency (raw value, i.e., correct responses; decrease in dual condition; cluster size: number of switches; percentage of correct responses produced between 0–15″ and 46–60″) and visuomotor interfering task (raw value, i.e., correct responses; decrease) measurements according to single and dual condition.

	Single condition		Speed interference		Semantic interference		Phonological interference		Flexibility interference	
Fluency	Letter	Semantic	Letter	Semantic	Letter	Semantic	Letter	Semantic	Letter	Semantic
Raw value	15.2 ± 4.9	21.1 ± 5.9	10.9 ± 4.2	14.3 ± 4.6	11.1 ± 4.7	13.1 ± 4.6	7.9 ± 3.8	10.1 ± 4.1	9.9 ± 4.0	12.0 ± 4.1
Decrease (%)			−26.5 ± 22	−30.5 ± 19	−25.5 ± 28	−36.6 ± 21	−46.9 ± 24	−51.0 ± 20	−32.4 ± 25	−41.5 ± 20
Cluster size	0.46 ± 0.4	2.10 ± 1.3	0.41 ± 0.4	1.95 ± 1.6	0.37 ± 0.3	1.82 ± 1.5	0.37 ± 0.4	1.49 ± 1.0	0.44 ± 0.4	1.81 ± 1.6
Switch (n)	10.5 ± 3.9	1.50 ± 1.0	7.77 ± 3.5	5.04 ± 2.5	8.05 ± 3.9	4.78 ± 2.6	5.36 ± 3.2	3.83 ± 2.3	6.80 ± 3.4	4.36 ± 2.4
0–15″ (%)	40.2 ± 12	40.1 ± 13	47.2 ± 16	42.9 ± 14	39.9 ± 16	38.0 ± 17	36.4 ± 18	35.7 ± 18	43.9 ± 19	40.7 ± 15
46–60″ (%)	17.5 ± 9	16.6 ± 10	14.3 ± 10	15.4 ± 10	16.3 ± 12	17.3 ± 12	17.4 ± 15	18.2 ± 13	14.8 ± 12	16.7 ± 11
Visuomotor										
Raw value	Supplementary Table A		17.6 ± 7	19.5 ± 7	18.1 ± 6	18.8 ± 6	9.0 ± 4	10.1 ± 4	12.2 ± 3	12.6 ± 3
Decrease (%)			−56.6 ± 17	−51.8 ± 16	−33.0 ± 20	−30.0 ± 22	−42.5 ± 23	−35.6 ± 26	−37.0 ± 17	−35.0 ± 16

These findings indicate that phonological interference has a prominent effect on the fluency task, while speed interference predominantly affects the visuomotor interfering task. Importantly, these results do not compromise the analysis of the decrement in verbal fluency task alone.

**Figure 4:**
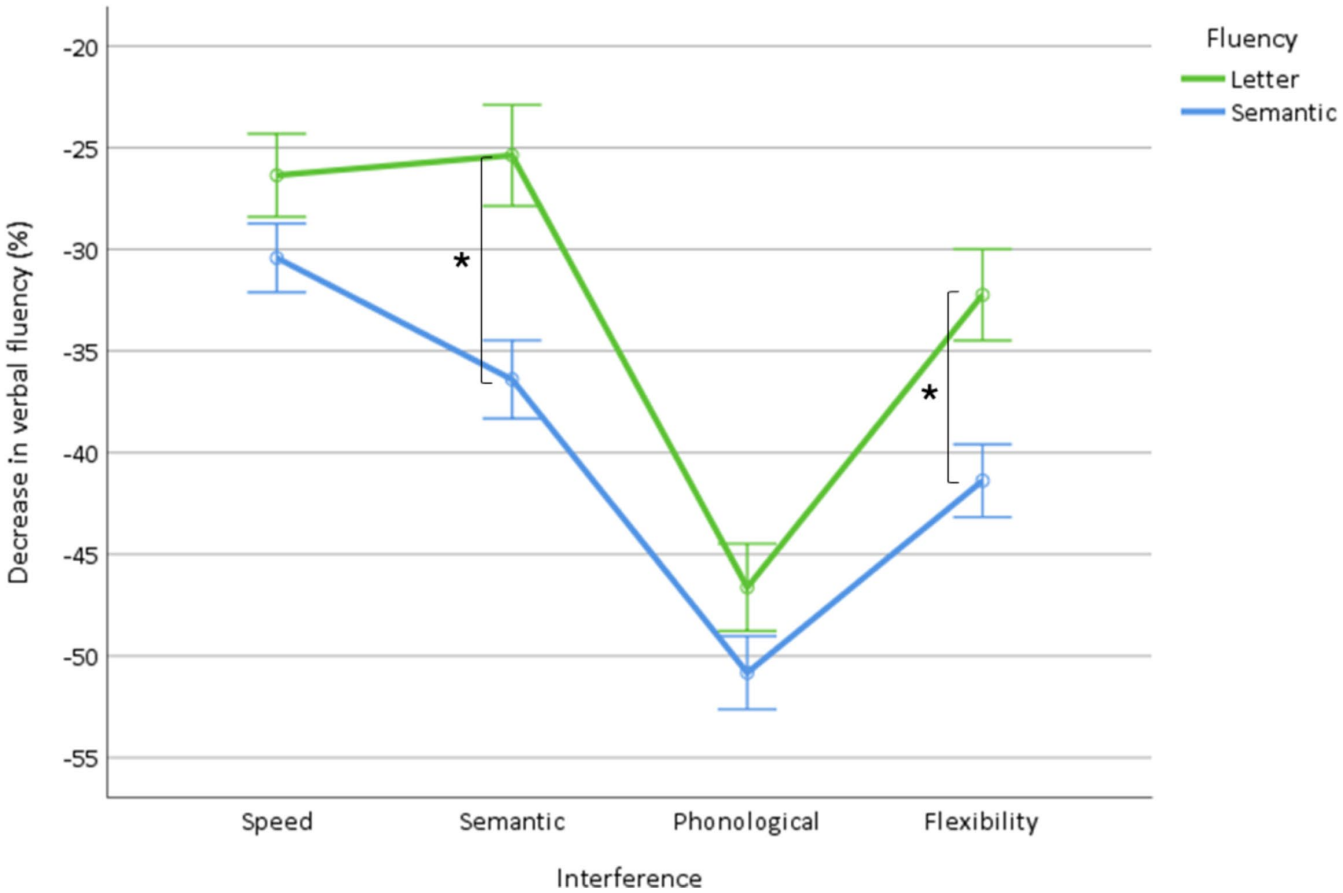
Decrease in fluency (dual condition minus single condition) according to the type of interference (speed, semantic, phonological, flexibility). *Indicate significant interactions.

The complementary analysis of the effect of interference on the decrease in fluency showed (1) a fluency effect (*p* < 0.0001) due to a greater decrease in semantic fluency (−39.8 ± 0.7) than letter fluency (−32.8 ± 0.9), (2) an interference effect (*p* < 0.0001) due to a greater decrease in the phonological (−48.9 ± 0.8), then flexibility (−36.9 ± 0.8), then semantic (−31.0 ± 0.9), and then speed (−28.5 ± 0.7) interference (*p* < 0.0001, all), and (3) a fluency × interference interaction (*p* < 0.0001, Figure 4, Table 2). The semantic (*p* < 0.0001) and flexibility (*p* < 0.0001) interference induced a greater decrease in semantic fluency, whereas the speed and the phonological and the interferences did not differ (*p* = 0.9) between the types of fluency.

Overall, these results mainly show that all visuomotor interfering tasks reduced fluency, with the greater decrease observed in phonological, followed by flexibility, semantic, and then speed interference. Additionally, the decrement was more pronounced in semantic fluency, with an even greater reduction under semantic interference and flexibility interference.

### 3.2 Effect of interference on clustering and switching

**Figure 5:**
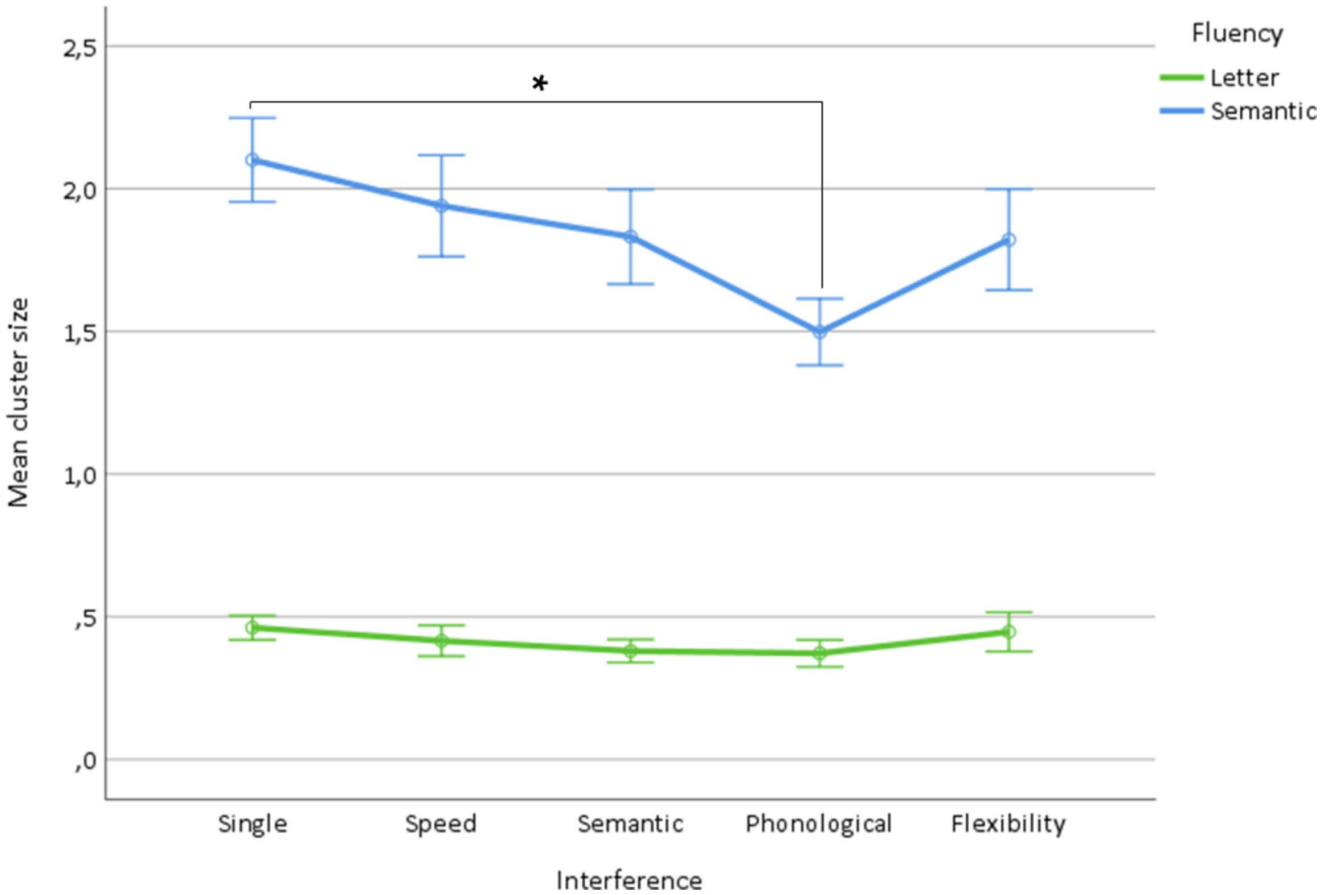
Mean cluster size for letter and semantic fluency as a function of the type of interference (single condition, speed, semantic, phonological, flexibility). *Indicate significant interactions.

The ANOVA on cluster size showed (1) a fluency effect (*p* < 0.0001) due to a higher cluster size in semantic fluency (letter: 0.42 ± 0.1, semantic: 1.83 ± 0.1), (2) an interference effect (*p* < 0.0001) due to a decrease in cluster size (single condition: 1.28 ± 0.1, speed interference: 1.18 ± 0.1) significant for the semantic (1.09 ± 0.1), phonological (0.93 ± 0.1), and flexibility (1.13 ± 0.1) interference, with a prominent decrease (*p* < 0.0001) for phonological interference, and (3) a fluency × interference interaction (*p* < 0.0001) related to the predominant decrease in cluster size for phonological interference for semantic fluency (Table 2 and Figure 5).

**Figure 6:**
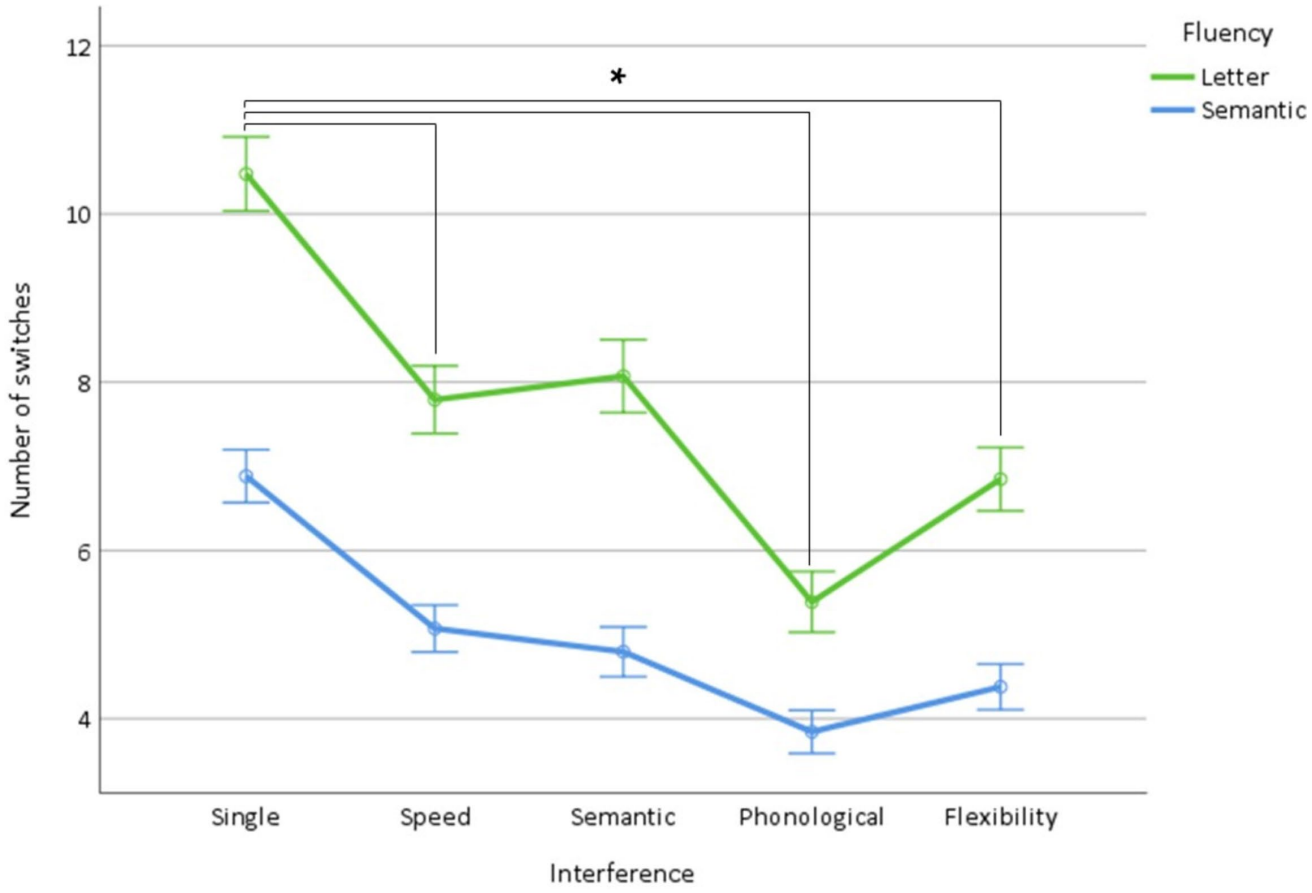
Number of switches in letter and semantic fluency as a function of interference (single condition, speed, semantic, phonological, flexibility). *Indicate significant interactions.

The ANOVA on the number of switches showed (1) a fluency effect (*p* < 0.0001) due to a higher number of switches in letter fluency (letter: 7.7 ± 0.2, semantic: 5.0 ± 0.1), (2) an interference effect (*p* < 0.0001) due to a decrease (*p* < 0.0001, all) in the number of switches for all interference types (single condition: 8.7 ± 0.1; speed: 6.4 ± 0.1; semantic: 6.4 ± 0.1; phonological: 4.5 ± 0.1; flexibility: 5.6 ± 0.1) that was prominent for phonological interference (*p* < 0.0001), followed by flexibility interference (*p* < 0.0001), and (3) a fluency × interference interaction (*p* < 0.0001) related to the prominent decrease of the number of switches in letter fluency for all types of interference (*p* < 0.001, all), except semantic interference (Table 2 and Figure 6).

Overall, these results indicate that the mean cluster size is affected by linguistic (i.e., phonological and semantic) and flexibility interference, but not by simple speed interference, whereas the number of switches is affected by all types of interference. In addition, they show that semantic fluency is characterized by greater cluster size, which is more sensitive to interference, and, conversely, that letter fluency is characterized by a higher number of switches, which is more sensitive to interference.

### 3.3 Effect of interference on the time course

**Figure 7:**
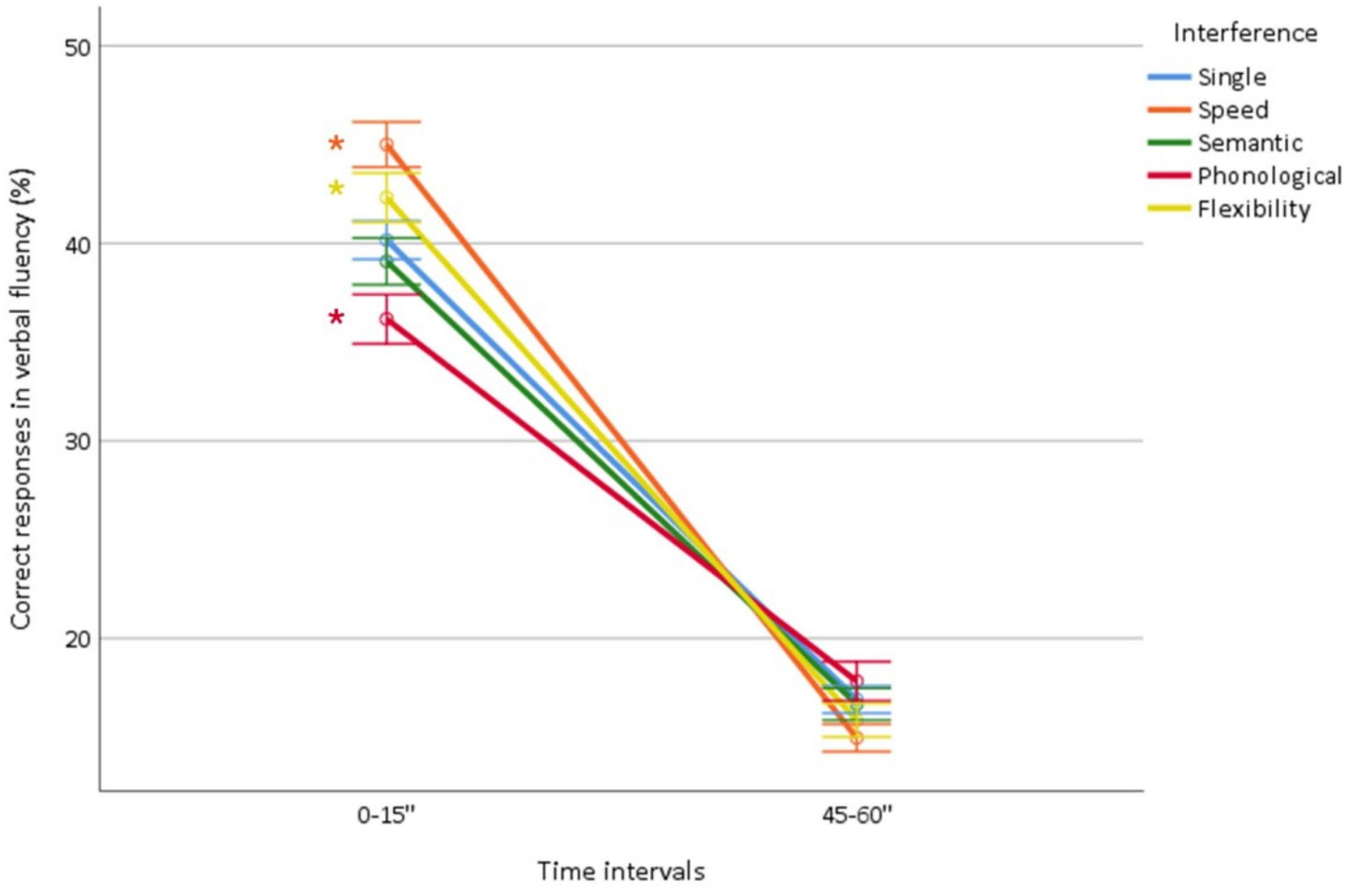
Percentage of correct responses produced in each time interval (0–15″; 45–60″) as a function of interference (single condition, speed, semantic, phonological, flexibility). *Indicate significant interactions.

The ANOVA of the percentage of words produced in 0–15″ and 45–60″ showed (1) a time course effect (*p* < 0.0001) due to an imbalance, with higher production in the first time-interval (0–15″: 37.3 ± 0.41%, 45–60″: 16.4 ± 0.3%), (2) a fluency effect (*p* = 0.004) due to higher production in letter fluency (letter: 28.8 ± 0.18%, semantic: 24.6 ± 0.14%), (3) an interference effect (*p* < 0.0001) due to lower production (*p* = 0.02, all) for semantic (27.8 ± 0.25%) and phonological (26.9 ± 0.29%) interference and higher production (*p* < 0.0001) for speed interference (30.0 ± 0.23%), whereas flexibility (29.0 ± 0.25%) did not differ from the single condition (28.5 ± 18%) (*p* = 0.051), (4) a time course × interference interaction (*p* < 0.0001) (Figure 7) related to an increased imbalance in favor of the first 15″ interval for speed and flexibility interference resulting in a higher percentage of correct responses compared to the simple condition from 0″ to 15″ and a lower percentage of correct responses compared to the simple condition from 46″ to 60″ (*p* < 0.0001, both) and a reduced imbalance in favor of the first 15″ interval for phonological interference, conversely resulting in a lower percentage of correct responses compared to the simple condition from 0″ to 15″ and a higher percentage of correct responses compared to the simple condition from 46″ to 60″ (*p* < 0.0001), whereas there was no difference between semantic interference and the single condition (*p* = 0.2), (5) a time course × fluency interaction (*p* < 0.0001) related to an increased imbalance in favor of the first 15″ interval in letter fluency (letter: 0–15″: 41.6 ± 0.5%, 45–60″: 16.0 ± 0.3%; semantic: 0–15″: 39.5 ± 0.5%, 45–60″: 16.9 ± 0.3%), (6) a fluency × interference interaction (*p* = 0.039) due to an increased imbalance in letter fluency with speed interference (*p* = 0.008) (letter fluency: single condition: 28.7 ± 0.2%, speed: 30.8 ± 0.3%, semantic: 28.1 ± 0.3%, phonological: 26.9 ± 0.4%, flexibility: 29.4 ± 0.4%; semantic fluency: single condition: 28.4 ± 0.2%, speed: 29.1 ± 0.3%, semantic: 27.6 ± 0.3%, phonological: 27.0 ± 0.4%, flexibility: 28.8 ± 0.3%), and (7) a time course × fluency × interference interaction (*p* < 0.0001) (Table 2; Supplementary Figure C) due to an increased imbalance in favor of the first 15″ interval in letter fluency with the speed and flexibility interference (*p* < 0.0001, both).

Overall, these results mainly indicate that the imbalance favoring word production in the first 15″ is more pronounced for executive (i.e., speed and flexibility) than linguistic (i.e., semantic and phonological) interference, and is greater for letter fluency.

## 4 Discussion

This study performed on a large control group documents the effect of linguistic and executive interference on letter and semantic fluency measured both at the raw level (i.e., number of correct responses) and at the level of derived indices (i.e., cluster size, number of switches, and time course of production). We found an overall decrease in word production induced by all types of interference, with the specific effect depending on the type of interference associated with consistent changes in the derived indices.

First, the interference induced by the simple concurrent task that only engages the attentional activation system (speed interference) decreased word production. There was no competition between the two tasks for common structural processes (i.e., the fluency task involved verbal output, whereas the interfering task involved visuomotor processes), thus implying that the decrease was due to competition for attentional resources (Fournet et al., 2021). This supports the prediction of our model (Figure 1) that a general attentional process, indexed by psychomotor speed, is involved in accelerating the completion of the verbal fluency task. Our conclusion is reinforced by a similar finding of decreased fluency during secondary simple motor and visuomotor tasks (Fournet et al., 2021; Moscovitch, 1994; Rende et al., 2002; Troyer et al., 1997). These findings obtained from an interventional design add a higher level of evidence to previous findings based on an association design, showing that psychomotor speed accounts for a significant part of the variance in verbal fluency (Godefroy et al., 2023; Gonzalez-Burgos et al., 2019; Kassir et al., 2023; Whiteside et al., 2016).

A second prediction of our model is that secondary tasks that engage attention and additional linguistic processes, such as phonological or semantic processes (semantic and phonological interference), would have a greater impact on verbal fluency than speed interference. The observed greater decrease in fluency induced by phonological and semantic interference relative to speed interference verifies this prediction.

A third prediction is that a concurrent task that mainly engages semantic memory through visual access (semantic interference) would have a greater impact on semantic fluency than letter fluency. Indeed, semantic interference induced a greater decrease in semantic fluency, thus verifying the prediction. This indicates that some degree of semantic processing is involved in semantic fluency, in accordance with the previous finding of a greater decrease in semantic fluency induced by a concurrent object decision task designed to require the activation of semantic knowledge (Martin et al., 1994).

A fourth prediction of our model is that a concurrent task that primarily engages the phonological output lexicon and, more generally, the sequential chain of oral production (phonological interference) should have a major impact on both fluency tasks. The decrease in fluency induced by phonological interference was the greatest and was observed for both letter and semantic fluency, thus verifying the prediction. This finding converges with that of a previous dual-task study (Rende et al., 2002) and supports that phonological processes are involved in both verbal fluency tasks.

Fifth, we had no prediction concerning the effect of flexibility interference. This condition requires performing the visuomotor task in ascending order, while alternating colors, and is known to be difficult, involving flexibility. This visuomotor interfering task can be linked to previously used secondary tasks involving switching or inhibition (Fournet et al., 2021; Rende et al., 2002). Although the ability to alternate between categories is not a process involved in the fluency task according to our model, this difficult task and verbal fluency tasks could compete for certain common executive resources. Executive processes are now more frequently considered to be distinct, i.e., dependent on different, although close, brain structures (Godefroy et al., 2018, 2024; Stuss et al., 2005). However, the decrease induced by flexibility interference was very high, ranking second after phonological interference. This finding supports that both fluency and alternating tasks compete for certain common executive resources and warrants further investigation.

Overall, these results support our model by indicating that the fluency task involves lexico-phonological and semantic processes with which the strategic search process interacts, as well as an attentional component necessary to accelerate overall processing. These results also indicate interactions with other executive processes, such as those involved in stimulus dimension alternation, and require further analysis.

The present results also contribute to identifying the processes that are assessed by derived indices, i.e., cluster size, number of switches, and time course of word production. Concerning cluster size and the number of switches, we did not find a double dissociation, i.e., a specific decrease in cluster size induced by linguistic interference and a specific decrease in the number of switches induced by executive interference. This finding suggests that these indices do not purely reflect linguistic and executive processes, respectively. However, the lack of a decrease in cluster size induced by speed interference supports that clustering is independent of attention and psychomotor speed. Conversely, the number of switches was sensitive to speed interference, thus supporting that switching is sensitive to attentional allocation. This finding supports previous observations obtained using dual tasks (Rende et al., 2002; Troyer et al., 1997) and association designs (Unsworth et al., 2011). This result only very partially confirms the standard interpretation of cluster size in terms of the linguistic index and the number of switches in terms of the executive index (Bose et al., 2022; Faroqi-Shah and Milman, 2018; Troyer et al., 1997, 1998).

The interpretation of the time course of word production is still debated. Some studies have suggested that initial production reflects linguistic processes and final production, executive processes (Bose et al., 2022; Luo et al., 2010; Michalko et al., 2023), whereas the opposite has been suggested by others (Kassir et al., 2023; Mayr and Kliegl, 2000; Raboutet et al., 2010). Our results do not provide evidence for a reversal of the imbalance between the initial (major) and final (minor) 15″ of production. However, the finding that phonological interference attenuated the imbalance, i.e., decreased mainly the production in 0–15″, strongly supports that initial production reflects prominently linguistic processes. Reciprocally, the finding that both types of executive interference (i.e., speed and flexibility) reinforced the imbalance, i.e., decreased mainly the production in 46–60″, strongly supports that final production reflects prominently executive processes (strategic search and processing speed). This supports the prominent contribution of automatic lexical retrieval processes in the initial phase of fluency and, as the task progresses over time, the progressively increased contribution of executive processes (Bose et al., 2022; Friesen et al., 2015; Michalko et al., 2023; Shao et al., 2014). Importantly, our interference design offers robust evidence, surpassing the limitations of previous studies with lower levels of evidence, such as association designs confounded by additional factors (Michalko et al., 2023) or lacking confirmation through correlation analyses (Bose et al., 2022). The lack of a time effect of semantic interference should be interpreted with caution. However, it indicates that the decrease in word production induced by semantic interference does not affect the time course of word production. This finding suggests the overall involvement of semantic processes throughout the fluency task, regardless of the time point.

Our study had several limitations. First, it assessed the validity of our model using interference tasks focusing on linguistic and attentional components but not the key strategic search process. However, we were unable to find a secondary task involving the strategic word search process that resulted in a feasible dual task. Although visuomotor interfering tasks require a certain degree of spatial target searching, they are unlikely to depend on the same process as word searches, which primarily require word activation based on phonological or semantic cues. Second, we did not compare the present results with those of more conventional executive and language tests. We carried out these tests but the results cannot be presented here due to lack of space. They will be presented in a forthcoming paper, together with other conditions designed to more specifically assess the word-finding process. Third, we did not assess the anatomical correlates of the processes in this study. In addition to previous studies (Godefroy et al., 2023, 2024), this aspect will be the focus of a future study carried out on patients.

Our study had several strengths. Firstly, the present study is the only one to have tested a fluency task model using dual-task paradigms that interfere with speed, semantic, phonological, and flexibility processes with both letter and semantic fluency. Findings obtained using an interference design, which constitutes an intervention paradigm, provide a higher level of evidence. Secondly, this is the first study to assess such a large number of fluency indices on a very large sample of 487 healthy participants. Thirdly, we provide findings consistent with those of previous studies that contribute to the modeling of fluency and the interpretation of derived indices.

## 5 Conclusion

The present results, based on an interference design, support that the semantic and output lexico-phonological processes involved in the fluency task interact with an attentional system (necessary to optimize overall task performance) and an executive process corresponding to the strategic search process in our model. Additionally, these results highlight interactions with other executive functions, such as those involved in alternating, which warrant further investigation. Our findings provide empirical support for our model (Godefroy et al., 2023) and offer valuable insights into derived indices; the commonly cited associations between executive functioning and switching, as well as between semantic ability and clustering, are only partially supported. Moreover, word production appears to be influenced by different cognitive processes over time, with early production driven by the phonological output lexicon, while later production requires more complex executive processing.

From a conceptual point of view, verbal fluency tasks provide a unique opportunity to explore the functional architecture of control functions by revealing the interactions between executive processes and lower-level processes, such as language. From a clinical point of view, by clarifying the functional architecture of verbal fluency—specifically the contributions and interactions between linguistic and executive processes—this work enables practitioners to more accurately identify the cause of verbal fluency impairments, leading to more precise cognitive diagnoses and more effective rehabilitation.

## Data Availability

The raw data supporting the conclusions of this article will be made available by the authors, without undue reservation. “Readers seeking access to full data should contact the corresponding author (Flore Dorchies) at the Laboratory of Functional Neurosciences (UR UPJV 4559) (Amiens, France)”.

## Footnotes

^1^ www.cebm.net
