# Supplementary material for: Investigating the cognitive architecture of verbal fluency: evidence from an interference design on 487 controls
Source: Front Psychol. 2024 Dec 16;15:1441023. doi: 10.3389/fpsyg.2024.1441023 (PMC11683421; doi:10.3389/fpsyg.2024.1441023)
Supplement: Supplementary file 1 [file Presentation_1.pdf]

Online Supplement

1. Exclusion criteria

- Non-French speaking: foreign mother tongue
- Unsigned information letter
- Resident in an institution (retirement home, etc.)
- Cannot read
- Cannot count to 36 or does not know the alphabet
- Visual or auditory deficits that interfere with test-taking
- Average consumption of more than three glasses of alcohol per day
- History of alcohol withdrawal
- Use of drugs for less than three months
- History of drug withdrawal syndrome
- General anesthesia less than three months prior
- History of cardiac surgery with extracorporeal circulation
- Current or past cerebral pathology: coma or loss of consciousness lasting more than 15 minutes, head trauma with loss of consciousness lasting more than 15 minutes, stroke or hemiplegia or aphasia, neurological follow-up for cognitive disorders (memory, language, etc.), neurological follow-up for neurodegenerative disease (Parkinson's disease, multiple sclerosis, etc.), epilepsy requiring current treatment, cerebral radiotherapy
- Psychiatric pathology (including depression): requiring current treatment, having required a stay of more than two days in a specialized environment, anxiety currently requiring more than one medication, current antidepressant treatment, current antiepileptic treatment, anxiolytic treatment initiated or increased in the last month

Wearing glasses and/or using hearing aids were not exclusion criteria. It was only necessary to ensure that participants wore/used them during the test.

2. Cognitive assessment diagram

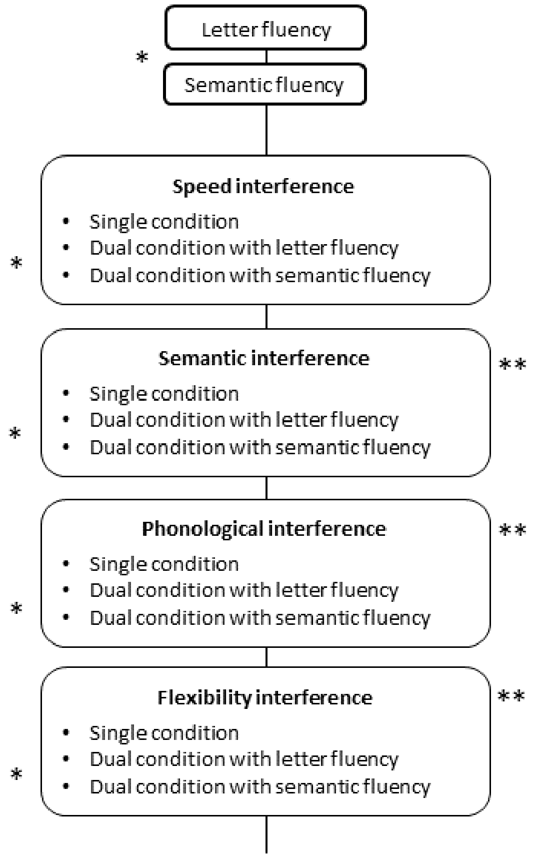

Supplement Figure A. Diagram of the cognitive assessment. *Note.* We counterbalanced (*) fluency: order A, letter>semantic; order B, semantic>letter, and (**) three types of interference (P, phonological, S, semantic, F, flexibility): order 1, P>S>F; order 2, P>F>S; order 3, S>P>F; order 4, S>F>P; order 5, F>P>S; order 6, F>S>P. This diagram represents the order A1.

3. Visuomotor interfering tasks performed in the single condition

Supplement Table A. Mean (± standard error) of visuomotor interfering tasks performed in the single condition.

	Speed interference	Semantic interference	Phonological interference	Flexibility interference
Single condition	42.9±15	27.4±6	16.6±5	20.1±6

4. Decrease in fluency and visuomotor interfering tasks

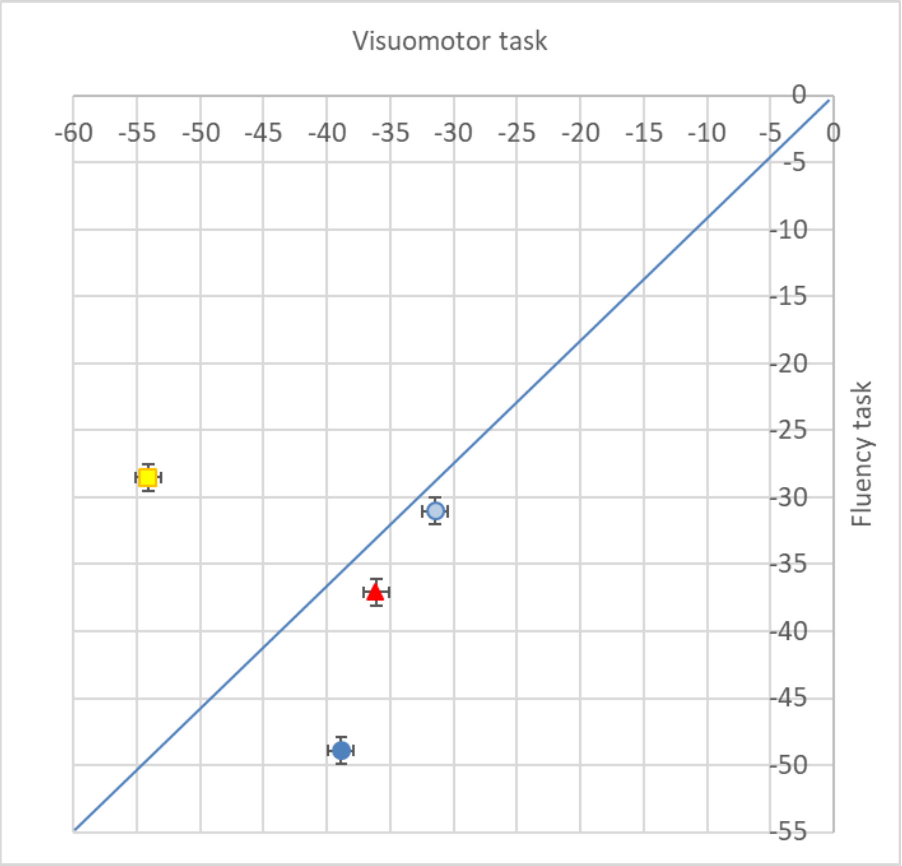

Supplement Figure B. Mean decrease (± standard error) in fluency (y axis) and visuomotor interfering (x axis) tasks as a function of interference (yellow: speed, light blue: semantic, deep blue: phonological, red: flexibility).

This graph shows a greater decrease induced by the speed interference in the visuomotor interfering task and a greater decrease induced by the phonological interference in the fluency task, whereas semantic and flexibility interference induced a similar decrease in both tasks (values close to the diagonal indicate a balanced decrease between the two tasks).

5. Imbalance between fluency production in the first and last 15’’

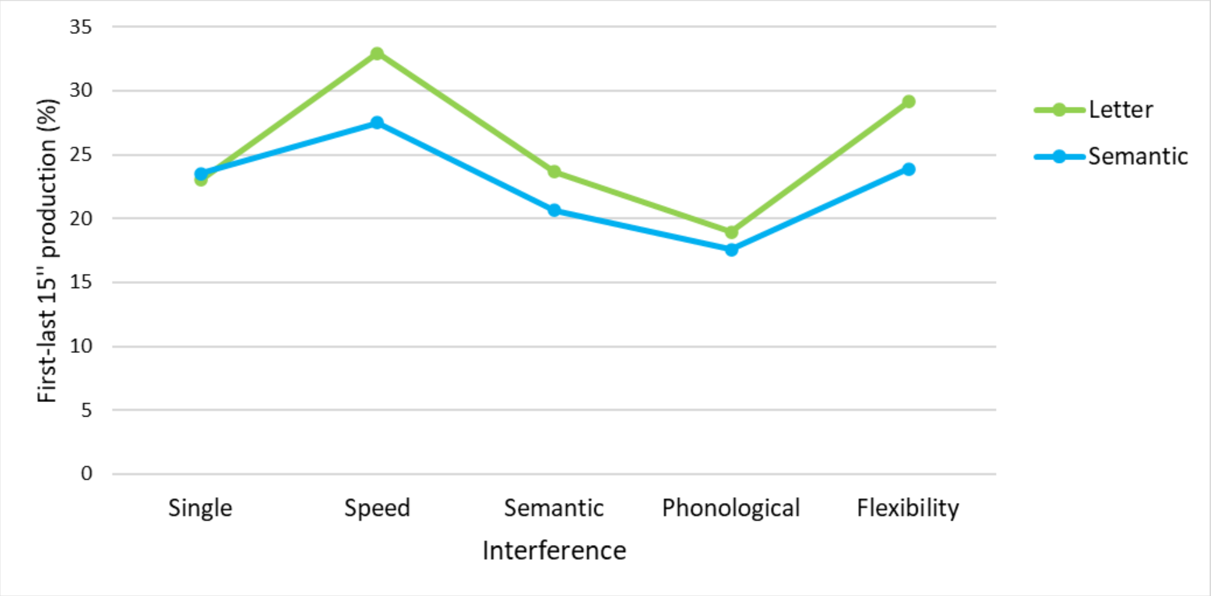

Supplement Figure C. Imbalance between production in the first and last 15'' (percentage of words produced in the first 15’’ minus percentage of words produced in the last 15’’) in letter and semantic fluency as a function of the type of interference (single condition, speed, semantic, phonological, flexibility).
